# Disparities in safe sex counseling & behavior among individuals with substance dependence: a cross-sectional study

**DOI:** 10.1186/1742-4755-9-35

**Published:** 2012-12-31

**Authors:** Meredith M D’Amore, Debbie M Cheng, Donald Allensworth-Davies, Jeffrey H Samet, Richard Saitz

**Affiliations:** 1 Health/care Disparities Research Program, Section of General Internal Medicine, Boston University School of Medicine, Boston Medical Center, 801 Massachusetts Avenue, #2098, Boston, MA, 02118, USA; 2 Clinical Addiction Research and Education (CARE) Unit, Section of General Internal Medicine, Department of Medicine, Boston University School of Medicine and Boston Medical Center, Boston, MA, 02118, USA; 3 Department of Biostatistics, Boston University School of Public Health, Boston, MA, 02118, USA; 4 School of Health Sciences, Cleveland State University, Cleveland, OH, 44115, USA; 5 Department of Community Health Sciences, Boston University School of Public Health, Boston, MA, 02118, USA; 6 Department of Epidemiology, Boston University School of Public Health, Boston, MA, 02118, USA

**Keywords:** Counseling, Disparities, Sexual behavior, Stereotyping

## Abstract

**Background:**

Despite the vast literature examining disparities in medical care, little is known about racial/ethnic and mental health disparities in sexual health care. The objective of this study was to assess disparities in safe sex counseling and resultant behavior among a patient population at risk of negative sexual health outcomes.

**Methods:**

We conducted a cross-sectional analysis among a sample of substance dependent men and women in a metropolitan area in the United States. Multiple logistic regression models were used to explore the relationship between race/ethnicity (non-Hispanic black; Hispanic; non-Hispanic white) and three indicators of mental illness (moderately severe to severe depression; any manic episodes; ≥3 psychotic symptoms) with two self-reported outcomes: receipt of safe sex counseling from a primary care physician and having practiced safer sex because of counseling.

**Results:**

Among 275 substance-dependent adults, approximately 71% (195/275) reported ever being counseled by their regular doctor about safe sex. Among these 195 subjects, 76% (149/195) reported practicing safer sex because of this advice. Blacks (adjusted odds ratio (AOR): 2.71; 95% confidence interval (CI): 1.36,5.42) and those reporting manic episodes (AOR: 2.41; 95% CI: 1.26,4.60) had higher odds of safe sex counseling. Neither race/ethnicity nor any indicator of mental illness was significantly associated with practicing safer sex because of counseling.

**Conclusions:**

Those with past manic episodes reported more safe sex counseling, which is appropriate given that hypersexuality is a known symptom of mania. Black patients reported more safe sex counseling than white patients, despite controlling for sexual risk. One potential explanation is that counseling was conducted based on assumptions about sexual risk behaviors and patient race. There were no significant disparities in self-reported safer sex practices because of counseling, suggesting that increased counseling did not differentially affect safe sex behavior for black patients and those with manic episodes. Exploring the basis of how patient characteristics can influence counseling and resultant behavior merits further exploration to help reduce disparities in safe sex counseling and outcomes.

**Trial registration:**

NCT00278447

## Background

Sexually transmitted infections (STIs) and unintended pregnancy are prevalent among racial/ethnic minorities and individuals with mental illness, and associated with a host of negative health outcomes and costs [1-7]. Unintended pregnancy is costly on many levels, including direct medical costs of births, abortions and fetal losses, indirect costs of wages lost from not working and psychological costs associated with the challenges posed by unintended pregnancy [8-10]. Unintended pregnancies were recently estimated to cost taxpayers $11 billion each year [11,12]. Unintended pregnancies affect the parents (who may endure financial hardship and limitations to their educational attainment) [9,10] and children, in terms of birth outcomes and worse cognitive, emotional and behavioral development [9,10,13-15]. Sexually transmitted infections, including chlamydia, gonorrhea, human papiloma virus (HPV) and the human immunodeficiency virus (HIV), are highly prevalent, preventable infections that can lead to serious health consequences including chronic pain, infertility and mortality [1]. The costs associated with the treatment of STIs are substantial, with an estimated $6.5 billion expended in 2000, for 15-24 year olds alone [16].

In 2001, the rate of unintended pregnancy was highest for black and Hispanic women (98 and 78 per 1,000, respectively), compared with that for whites (35 per 1,000) [3]. More recent data from 2006-2010 report the following percentages of unintended pregnancies resulting in births: 20% non-Hispanic white, 35% Hispanic and 45% black women [17]. Disparities also exist in rates of STIs. For example, black men and women are most affected by chlamydia and gonorrhea, having 9-19 times higher rates than whites [1]. Patients with mental illness have worse health outcomes and a higher medical burden compared to the general population [18-20], including high rates of unintended pregnancy and abortion [5,21,22]. Individuals with mental illness are at higher risk for acquiring STIs and having unintended pregnancies due to increased rates of unprotected intercourse as a result of having less knowledge about contraception, lower capacity to plan ahead, inability to navigate contraceptive resources and being at higher risk for sexual coercion [4-6,23-32].

Substance use, the consumption of alcohol and/or use of illicit drugs, is also associated with sexual risk behaviors [7,33]. The precise link between substance use and engagement in sexual risk behaviors has not been fully established in the literature, but studies suggest that this relationship is primarily a function of less consistent condom use and having multiple sex partners [7,34,35]. Thus, individuals with substance dependence are another vulnerable population at increased risk for unprotected sex, unintended pregnancy and STIs [2].

Identifying barriers to contraceptive usage and safe sexual behaviors is vital in order to prevent STIs and unintended pregnancy. Clinicians can play a pivotal role in educating patients about safe sex and helping to increase their knowledge and use of contraception [36-39]. Although many factors outside of the medical encounter can influence patient sexual behavior and contraception use, clinicians have an opportunity to counsel populations at risk of negative sexual health outcomes, with the potential of affecting patient behavior. This counseling has the potential to reduce risky sexual behaviors and related negative health outcomes [36-41]. Contraceptive counseling in primary care, where there is a preventive focus and a longitudinal relationship with patients, can impact patient contraceptive use and method choice [42].

Despite a higher prevalence of sexual risk behaviors among people with substance dependence than in the general population [7], little is known about disparities in sexual health care among these individuals. Communication problems in the patient-clinician exchange may occur differentially across race/ethnicities and among patients with mental illness, thereby contributing to the disparities in contraceptive use and, subsequently, STIs and unintended pregnancy [37,43]. Primary care clinicians in particular may feel discomfort in discussing these issues because of their sensitive nature and lack of training [44-46]. Despite the vast literature about racial disparities in medical care, little is known about disparities specific to sexual health care [47]. One recent study of low income women found that blacks were more likely to report being pressured by their clinician to use contraceptives, compared to whites [47]. A series of studies found that many African Americans who received family planning care felt discriminated against and held conspiracy beliefs about birth control (such as “birth control is a form of Black genocide”) [48].

There is also a dearth of literature assessing disparities in clinician safe sex counseling for people with mental illness [49]. Clinicians may unknowingly make assumptions about the sexual risks of patients with mental illness or their ability to comply with birth control regimens, which may affect clinicians’ decisions to provide appropriate counseling and discuss contraceptive options. Sexual health counseling for patients with mental illness is complex and challenging. Clinicians have the challenge of assessing the patient’s autonomy in decision-making and risk of unintended pregnancy and STIs. They also need to use judgment to consider how capable the patient is of using contraceptives consistently and effectively [29]. They need to ensure patients fully understand contraceptive options, risks, and benefits [29]. Guidelines to help clinicians assess patient autonomy may be subverted by subconscious bias in decision-making. The role clinicians can play in addressing the sexual health needs of individuals with mental illness warrants further study [50].

Further investigation into the role of clinicians is essential to improving disparities in patients’ safe sex behaviors, including the usage of and adherence to contraception. The objectives of this study are therefore to examine whether race/ethnicity and indicators of mental illness are associated with two separate outcomes: patient report of primary clinician’s safe sex counseling and practicing safe sex due to counseling among individuals with substance dependence. We hypothesized that minority patients and those with serious mental illness symptoms may receive less safe sex counseling, but anticipate that these disparities will be attenuated after controlling for other patient sociodemographic characteristics, sexual risk behavior and the quality of clinician-patient relationship.

## Methods

This was a secondary analysis of cross-sectional data collected for a randomized controlled trial (Addiction Health Evaluation and Disease management (AHEAD) Study) conducted in Boston, Massachusetts from September 2006 to September 2008. This study was approved by the Boston University Medical Campus Institutional Review Board (H-23464). All subjects provided informed consent, and procedures were followed in accordance with the Helsinki Declaration of 1975. A certificate of confidentiality was obtained from the National Institute on Alcohol Abuse and Alcoholism to further protect participants' privacy. The AHEAD study is a randomized clinical trial evaluating chronic disease management for substance dependence in primary care. All subjects had current alcohol and/or drug dependence, by DSM IV criteria [51] (assessed using the Composite International Diagnostic Interview Short Form) [52], were willing to establish or continue primary care at the study location and had engaged in recent heavy drinking or recent drug use. If they had primary care elsewhere but wanted to change to Boston Medical Center (BMC), they were considered eligible. If they had no primary care clinician, they had to be willing to be referred to one at BMC. These subjects were primarily recruited from a residential detoxification unit, but also from a large urban safety-net primary care clinic and through recruitment advertisements on public transportation. Subjects were at least 18 years of age, spoke English or Spanish, and were without indication of cognitive impairment at screening (assessed by a Mini Mental State Examination score greater than 20) [53]. Half of the enrolled subjects were randomized to the AHEAD clinic intervention, which included a team comprised of a nurse care manager, social worker, psychiatrist and internist. All study subjects were reimbursed $35 for completing all baseline visit procedures and $50 at the three month follow-up visit.

The inclusion criteria for our analysis included: completing the 3-month follow-up visit, reported having one particular doctor that they considered to be their regular primary care doctor and being self-reported non-Hispanic black, non-Hispanic white or Hispanic. The question about having a regular doctor asked: “Is there one particular doctor (or primary care provider, e.g. Nurse Practitioner or Physician’s Assistant) that you consider to be your regular personal primary care doctor?” Because this survey then continued to use the term “doctor” to encompass all of the primary care clinicians listed above, we continue to use the term “doctor” in reporting the results.

### Independent variables

The key independent variables in this study are race/ethnicity and three indicators of serious mental illness. Race/ethnicity was self-reported and categorized as: non-Hispanic white, non-Hispanic black, and Hispanic at the initial study visit. The number of subjects in other racial categories was small (n=20) and therefore were excluded from analyses. The three mental illness variables in this analysis include: moderately severe to severe depression; any past manic episodes; and ≥3 psychotic symptoms, all assessed at the three month study visit. (Mental illness was captured at baseline, but because most participants were starting a detoxification program the 3 month visit provided more accurate data.) Depression was assessed using the Patient Health Questionnaire short form (PHQ-9) which is comprised of nine items about respondents feelings in the last two weeks, such as “feeling down, depressed or hopeless”, with responses ranging on a four point scale from “not at all” to “nearly every day” (scores 0-3 points) [54]. We considered someone to be depressed if s/he had a PHQ-9 score of 15 or more, indicating moderately severe to severe depression [54,55]. Past manic or hypomanic episodes were assessed using the Mini International Neuropsychiatric Interview (MINI) [56]. Symptoms of mania include hypersexuality and impulsivity, and thus create the potential for increased sexual risk behavior [57]. This measure used a series of items to assess hypo/mania, asking about the frequency, duration and characteristics of manic episodes, with dichotomous responses of “Yes” or “No”. The MINI has been validated as a diagnostic tool in accordance with criteria from to the DSM-IV [56]. Psychotic symptoms were measured using four items from the Behavior and Symptom Identification Scale (BASIS) [58]. These items included: thinking you had special powers, hearing voices or seeing things, thinking people were watching you and thinking people were against you. Subjects were asked about their experience of these symptoms during the past week and asked to rate the frequency of these experiences on a scale ranging from “never” to “always”. We considered an individual to have substantial current psychotic symptoms if s/he responded “sometimes”, “often”, or “always” on three out of the four items. The complete BASIS measure is comprised of 24 items and using three out of four items not considered diagnostic, but an indication of psychosis.

### Dependent variables

The key dependent variables were two items about safe sex counseling and self-attributed resultant patient safe sex behavior, assessed during the three month study interview. The first item, having ever talked about safe sex with your regular doctor, was taken from the Primary Care Assessment Survey (PCAS) [59,60]. The PCAS is a patient reported instrument created to assess several domains that constitute quality primary care [59]. The specific question asked was “Which of the following has your regular doctor ever talked to you about:….safe sex?” (response: yes/no). The second dependent variable, having ever practiced safer sex because of your doctor’s advice, was taken from a survey used to examine the relationship between patient income and physician counseling about health risk behaviors [61]. The specific question asked was “Which of the following have you ever done because of your doctor’s advice?…Practiced safer sex” (response: yes/no). We included all patients who responded to these questions in our analysis. For the second dependent variable, having practiced safer sex because of a doctor’s advice, the sample was restricted to only those who had reported ever receiving safe sex counseling.

### Covariates

The covariates included in the analyses were self-reported age, gender, education (less than high school; high school; more than high school) and which randomized group the subject was assigned to, taken from the initial study visit. We also included whether the subject had multiple ( i.e., >1) male and/or female sex partners in the last three months, as an indication of sexual risk behavior. These data were taken from the audio computer assisted self-interviewing (ACASI) portion of the three month follow-up interview. We included these covariates given their potential effect on receipt of safe sex counseling and behavior [62-64]. We also included quality of the patient-doctor relationship, specifically assessing trust and communication from the PCAS, to evaluate if these variables attenuated any observed relationships between race/ethnicity, mental illness, and the outcomes of interest. The PCAS trust scale was scored based on a series of eight items and the communication scale was based on seven items [59]. These scales were scored as continuous variables and transformed to a scale of 0-100 for multivariable analyses [59].

### Statistical analysis

First, descriptive statistics were obtained for all variables stratified by each dependent variable (ever received safe sex counseling and ever practiced safer sex because of this advice). Bivariable tests were also performed for descriptive purposes. Next, we performed a series of multiple logistic regression models to test associations between indicators of mental illness and race/ethnicity with each outcome. For the outcome, having practiced safer sex because of a doctor’s advice, the analysis was restricted to the subset who reported ever receiving safe sex counseling. Spearman correlations were used to evaluate potential collinearity between independent variables and covariates. No pair of variables included in the same regression model was highly correlated (r>0.40). The following four models were fit for each of the two dependent variables. Model 1 was a preliminary, minimally adjusted model that included the main independent variables race/ethnicity and indicators of mental illness (depression, having had any manic episodes and psychotic symptoms), and two potential confounders: randomization group and multiple sex partners in the last three months. Model 2 additionally controlled for key sociodemographic characteristics: age, education, and gender. Model 3 added the trust scale from the PCAS as a continuous variable. Model 4, the final model representing the primary analyses, included the communication scale from PCAS as a continuous variable, and removed the trust scale. We did not include trust and communication scores in the same model due to their high correlation (r=0.67). The findings reported in the Results section are taken from the final model (Model 4) with communication score, unless otherwise specified. Adjusted odds ratios (AOR) and 95% confidence intervals (CI) are reported. All analyses were conducted using two-sided tests and a significance level of 0.05. Due to the exploratory nature of the analyses, adjustments were not made for multiple comparisons. Statistical analyses were performed by the Boston University School of Public Health’s Data Coordinating Center using SAS software (version 9.1; SAS Institute, Cary, NC).

## Results

Of the 563 individuals enrolled in the AHEAD study, 500 were followed up at 3 months. Of those, 299 reported that they had a regular doctor and 295 of those answered the two questions about safe sex counseling and behavior. After excluding the 20 subjects who were not black, white or Hispanic, our final sample for the analysis of ever received safe sex counseling included 275 individuals. The study sample was comprised of 44% white, 45% black and 11% Hispanic women and men (Table 1), with a mean age of 40 (range 18-61) and median age of 42 (not shown). Moderately severe to severe depression was common, reported among 58% of subjects. Forty percent reported a previous manic episode and 16% had at least three psychotic symptoms. Approximately 71% (195/275) of the sample reported having a doctor ever talk to them about safe sex. These 195 subjects comprised the sample for the analysis of ever practiced safer sex because of your doctor’s advice. Among the subset who received counseling, 76% (149/195) reported practicing safe sex because of their doctor’s advice. In the adjusted models, higher trust (AOR=1.03, 95% CI: 1.01, 1.04; Model 3) and better communication (AOR=1.02, 95% CI: 1.01, 1.04) were associated with higher odds of receipt of safe sex counseling (per 1 point increase on a scale of 0-100; Table 2). Having multiple sex partners in the last three months (AOR=0.44, 95% CI: 0.21, 0.94) was associated with lower odds of practicing safer sex because of this advice, compared to those with 0-1 sex partners (Table 3).

**Table 1:** Sample characteristics by receipt of safe sex counseling and practicing safer sex due to counseling

	**Doctor ever talked about safe sex n (%)**				**Ever practiced safe sex because of doctor’s advice n (%)**			
	**Yes**	**No**	**Total^a^**	**p-value^b^**	**Yes**	**No**	**Total^a^**	**p-value^b^**
	**(n=195)**	**(n=80)**	**(n=275)**		**(n=149)**	**(n=46)**	**(n=195)**	
Race/ethnicity								
Non-Hispanic white	74 (61.2)	47 (38.8)	121	**0.007**	51 (68.9)	23 (31.1)	74	0.12
Non-Hispanic black	97 (78.9)	26 (21.1)	123		77 (79.4)	20 (20.6)	97	
Hispanic	24 (77.4)	7 (22.6)	31		21 (87.5)	3 (12.5)	24	
Moderately severe to Severe depression (PHQ-9 score of ≥ 15)								
Yes	114 (71.7)	45 (28.3)	159	0.82	89 (78.1)	25 (21.9)	114	0.52
No	81 (70.4)	34 (29.6)	115		60 (74.1)	21 (25.9)	81	
Any manic or hypomanic episode								
Yes	88 (80.0)	22 (20.0)	110	**0.007**	67 (76.1)	21 (23.9)	88	0.94
No	107 (64.8)	58 (35.2)	165		82 (76.6)	25 (23.4)	107	
Three or more psychotic symptoms^c^								
Yes	36 (83.7)	7 (16.3)	43	**0.04**	31 (86.1)	5 (13.9)	36	0.19
No	159 (68.5)	73 (31.5)	232		118 (74.2)	41 (25.8)	159	
Age (years)								
<30	37 (69.8)	16 (30.2)	53	0.82	27 (73.0)	10 (27.0)	37	0.3
31-39	43 (72.9)	16 (27.1)	59		32 (74.4)	11 (25.6)	43	
40-49	80 (72.7)	30 (27.3)	110		59 (73.8)	21 (26.2)	80	
≥50	35 (66.0)	18 (34.0)	53		31 (88.6)	4 (11.4)	35	
Gender								
Female	70 (79.6)	18 (20.4)	88	**0.03**	55 (78.6)	15 (21.4)	70	0.6
Male	125 (66.8)	62 (33.2)	187		94 (75.2)	31 (24.8)	125	
Education								
Less than high school	49 (79.0)	13 (21.0)	62	0.28	39 (79.6)	10 (20.4)	49	0.82
High school graduate	91 (68.4)	42 (31.6)	133		69 (75.8)	22 (24.2)	91	
More than high school	55 (68.8)	25 (31.2)	80		41 (74.6)	14 (25.4)	55	
Randomization group								
Control	102 (72.3)	39 (27.7)	141	0.59	78 (76.5)	24 (23.5)	102	0.98
Intervention	93 (69.4)	41 (30.6)	134		71 (76.3)	22 (23.7)	93	
Multiple Sex Partners ^d^								
No	134 (71.7)	53 (28.3)	187	0.58	108 (80.6)	26 (19.4)	134	**0.03**
Yes	56 (68.3)	26 (31.7)	82		37 (66.1)	19 (33.9)	56	
PCAS trust score								
0-25	2 (66.7)	1 (33.3)	3	**0.02**	2 (100.0)	0 (0.0)	2	0.83
26-50	11 (45.8)	13 (54.2)	24		8 (72.7)	3 (27.3)	11	
51-75	91 (70.0)	39 (30.0)	130		67 (73.6)	24 (26.4)	91	
76-100	91 (77.1)	27 (22.9)	118		72 (79.1)	19 (20.9)	91	
PCAS communication score								
0-25	1 (33.3)	2 (66.7)	3	0.06	1 (100.0)	0 (0.0)	1	0.9
26-50	16 (57.1)	12 (42.9)	28		12 (75.0)	4 (25.0)	16	
51-75	68 (68.7)	31 (31.3)	99		51 (75.0)	17 (25.0)	68	
76-100	110 (76.4)	34 (23.6)	144		85 (77.3)	25 (22.7)	110	

^a^ missing responses not shown.

^b^ p-values calculated based on chi-square or Fisher’s exact tests as appropriate; p-values in bold are statistically significant (p<0.05).

^c^ Defined as responding “sometimes”, “often” or “always” on three out of four BASIS24 questions.

^d^ >1 sex partner within the past three months.

**Table 2:** Multivariable logistic regression models for receipt of safe sex counseling from primary care doctor

	**Doctor ever talked about safe sex**			
	**Model 1**	**Model 2**	**Model 3**	**Model 4**
	**AOR (95% Confidence interval)**			
***Main Independent Variables***				
Race/ethnicity				
Non-Hispanic black vs. white	2.38 (1.28, 4.44)^*^	2.82 (1.43, 5.56)^*^	2.78 (1.40, 5.56)^**^	2.71 (1.36, 5.42)^*^
Hispanic vs. white	1.87 (0.72, 4.87)	2.12 (0.77, 5.81)	2.58 (0.91, 7.26)	2.36 (0.83, 6.66)
Moderately severe to Severe depression (PHQ-9 score of ≥ 15)				
Yes vs. No	1.05 (0.59, 1.89)	1.02 (0.56, 1.86)	1.05 (0.57, 1.94)	0.98 (0.53, 1.82)
Any manic or hypomanic episode				
Yes vs. No	2.06 (1.12, 3.78)^*^	2.00 (1.08, 3.72)^*^	2.38 (1.25, 4.56)^**^	2.41 (1.26, 4.60)^**^
Three or more psychotic symptoms ^a^				
Yes vs. No	1.56 (0.62, 3.88)	1.80 (0.70, 4.58)	1.78 (0.68, 4.62)	1.78 (0.69, 4.62)
***Covariates***				
Randomization group				
Intervention vs. Control	0.86 (0.49, 1.49)	0.84 (0.48, 1.50)	0.87 (0.48, 1.57)	0.78 (0.43, 1.41)
Multiple Sex Partners (past 3 months)				
Yes vs. No	0.67 (0.37, 1.23)	0.65 (0.35, 1.22)	0.77 (0.41, 1.46)	0.77 (0.41, 1.45)
Age ^b^		0.97 (0.94, 1.00)	0.97 (0.94, 1.00)	0.97 (0.94, 1.00)
Gender				
Female vs. Male		1.85 (0.97, 3.55)	1.70 (0.87, 3.30)	1.74 (0.90, 3.38)
Education				
Less than high school vs. More than high school		1.24 (0.54, 2.85)	1.32 (0.56, 3.10)	1.45 (0.61, 3.46)
High school graduate vs. More than high school		0.85 (0.44, 1.63)	0.91 (0.47, 1.77)	0.92 (0.48, 1.79)
PCAS trust score ^c^			1.03 (1.01, 1.04)^**^	
PCAS communication score ^c^				1.02 (1.01, 1.04)^**^

^*^ p<0.05.

^**^ p<0.01.

^a^ Defined as responding “sometimes”, “often” or “always” on three out of four BASIS24 questions.

^b^ AORs associated with a 1 year increase in age.

^c^ AORs associated with a 1 point increase in PCAS scale score.

**Table 3:** Multivariable logistic regression models for practicing safer sex due to counseling from primary care doctor

	**Ever practiced safe sex because of doctor’s advice**			
	**Model 1**	**Model 2**	**Model 3**	**Model 4**
	**AOR (95% Confidence interval)**			
***Main Independent Variables***				
Race/ethnicity				
Non-Hispanic black vs. white	1.87 (0.88, 3.97)	1.84 (0.80, 4.22)	1.85 (0.81, 4.25)	1.85 (0.81, 4.26)
Hispanic vs. white	2.48 (0.65, 9.53)	2.56 (0.64, 10.19)	2.49 (0.63, 9.95)	2.56 (0.64, 10.17)
Moderately severe to Severe depression (PHQ-9 score of ≥ 15)				
Yes vs. No	1.26 (0.60, 2.65)	1.26 (0.60, 2.67)	1.25 (0.59, 2.65)	1.27 (0.60, 2.69)
Any manic or hypomanic episode				
Yes vs. No	0.84 (0.41, 1.74)	0.86 (0.41, 1.78)	0.82 (0.38, 1.74)	0.84 (0.39, 1.78)
Three or more psychotic symptoms ^a^				
Yes vs. No	2.05 (0.70, 6.07)	2.05 (0.68, 6.16)	2.06 (0.68, 6.18)	2.05 (0.68, 6.15)
***Covariates***				
Randomization group				
Intervention vs. Control	1.01 (0.50, 2.03)	1.01 (0.50, 2.06)	1.00 (0.49, 2.04)	1.01 (0.50, 2.06)
Multiple Sex Partners (past 3 months)				
Yes vs. No	0.43 (0.20, 0.89)^*^	0.44 (0.21, 0.94)^*^	0.44 (0.20, 0.93)^*^	0.44 (0.21, 0.94)^*^
Age ^b^		1.00 (0.96, 1.04)	1.00 (0.96, 1.04)	1.00 (0.96, 1.04)
Gender				
Female vs. Male		1.26 (0.58, 2.73)	1.31 (0.60, 2.88)	1.29 (0.59, 2.82)
Education				
Less than high school vs. More than high school		1.18 (0.43, 3.25)	1.15 (0.41, 3.18)	1.17 (0.42, 3.23)
High school graduate vs. More than high school		0.95 (0.41, 2.23)	0.92 (0.39, 2.18)	0.93 (0.39, 2.22)
PCAS trust score ^c^			0.99 (0.97, 1.02)	
PCAS communication score ^c^				1.00 (0.98, 1.02)

^*^ p<0.05.

^a^ Defined as responding “sometimes”, “often” or “always” on three out of four BASIS24 questions.

^b^ AORs associated with a 1 year increase in age.

^c^ AORs associated with a 1 point increase in PCAS scale score.

### Results by race/ethnicity

In bivariable analyses, whites had the lowest observed proportion of their doctor ever talking to them about safe sex (p=0.007; Table 1), compared to blacks or Hispanics. There were no significant findings by race/ethnicity for practicing safer sex because of a doctor’s advice. In the multivariable models, after adjustment for covariates, black subjects had significantly higher odds of a doctor having ever talked with them about safe sex (AOR=2.71, 95% CI: 1.36, 5.42; Table 2) compared to whites. No racial/ethnic group was associated with significantly higher odds of reporting ever practicing safe sex because of a doctor’s advice compared to whites (Table 3). Results were similar across all adjusted models.

### Results by indicators of mental illness

In bivariable analyses, those who reported a manic episode (p=0.007) or three or more psychotic symptoms (p=0.04) had higher odds of reporting that their doctor had ever talked to them about safe sex (Table 1), compared to those who had not had an episode or had less than three psychotic symptoms, respectively. None of the mental illness variables were significantly related to practicing safer sex because of a doctor’s advice. In fully adjusted models, having a manic episode remained significantly associated with higher odds of a doctor having ever talked to them about safe sex (AOR=2.41, 95% CI: 1.26, 4.60; Table 2). The association was statistically significant across all adjusted models. None of the mental health variables were significantly associated with practicing safer sex because of their doctor’s advice in adjusted models (Table 3).

## Discussion

The majority of adults with substance dependence in our sample reported being counseled about safe sex from their primary care doctor. Of those who were counseled, about three quarters reported that they practiced safer sex because of this advice. Our results suggest that improvements in safe sex counseling are needed for all individuals with substance dependence, particularly whites. This is consistent with prior literature that suggests general deficiencies in safe sex counseling and the need to integrate this into regular health care visits [41,61,65]. Lack of counseling may be a result of primary care clinicians feeling it is not their responsibility to counsel patients in sexual health behaviors [44]. Clinicians may also be uncomfortable in sexual health counseling and need more training related to sexual health [46]. Time constraints and lack of support staff have been cited as potential reasons for lack of sexual health counseling [44,45,66]. The expanded role of nurses or case managers may be a potential solution in improving quality sexual health care [45]. Of note, patient trust and communication with their doctor were the only covariates associated with receipt of safe sex counseling. This suggests that improving patient-doctor trust and communication may increase safe sex counseling for patients with substance dependence.

Our findings suggest that safe sex counseling can have a positive impact on patient behaviors. Understanding the discourse between patients and clinicians and effective methods for delivering contraceptive information is critical in developing practice guidelines and guidance for clinicians [67]. Future research should focus on understanding how clinicians can affect patient behavior, in an effort to reduce disparities in sexual health and outcomes.

Blacks and those with manic episodes reported being counseled by their doctors significantly more about safe sex. Although most who received counseling reported practicing safe sex because of their doctor’s advice, this was not more likely to translate into safer sex practices for one racial/ethnic group or individuals with indicators of mental illness. Interestingly, the covariates included in both sets of analyses largely did not affect the significant associations between race/ethnicity or mental illness with our outcomes. Although the findings that blacks are counseled more about safe sex are in contrast to our hypotheses, there is literature to support the finding of increased counseling for minorities [62]. Previous disparities research suggests that clinician behavior may be influenced by population based studies that affect their decision making in counseling patients [68,69]. Thus, if clinicians know from the literature that racial/ethnic minorities are more likely to have negative sexual health outcomes, they may counsel these patients more about safe sex practices. Clinicians may also have implicit, sub-conscious biases related to race that are not fully apparent but could affect patient counseling [70,71]. Counseling based on assumptions about sexual risk behaviors because of patient race can be considered stereotyping, that leads to disparities in counseling black patients more about safe sex. Instead, patient-centered counseling, based on individual risk factors, tailored to that individual’s needs, is ideal.

Patients with manic episodes may have been counseled more, given that hypersexuality is associated with these episodes [57]. It is difficult to disentangle the meaning of this finding, given that the subject was asked about current psychotic symptoms and depression, but *any* prior history of manic episodes. It is not clear if the manifestation of symptoms or diagnosis of mental illness came before or after safe sex counseling and thus we cannot determine if increased counseling among this group is in fact related to their mental illness. We also cannot verify that the clinician knew of the patient’s mental illness.

Although many interventions for increasing safe sex practices can be found in the literature [72], particularly for young and poor, minority women, there is not a major focus on individuals with substance dependence. Most of the interventions targeting substance users focus solely on reducing HIV infection [62,73], instead of prevention of general STIs and unintended pregnancy. Interventions should focus on the interaction of factors, such as substance use, race/ethnicity and mental illness, that may lead to disparities in sexual health outcomes [63]. Ultimately, reducing negative sexual health outcomes will require a multipronged intervention targeted at the patient, clinician and system level to help influence patient behavior and enhance the patient-clinician interaction [37].

Our findings should be interpreted within the limitations of our study. Although our analysis examined three racial/ethnic groups, we did not test for within group differences ( i.e., by type of Hispanic origin) or interactions by gender, because of potential sample size issues [74]. The findings that black patients and those with manic episodes had significantly higher odds of reporting being counseled could also be due to differential response bias or incomplete statistical adjustment (i.e., history of sexually transmitted infections). This study is based entirely on patient self-report, which may be subject to recall or social desirability bias. However, understanding if safe sex counseling occurred and how it affected behavior from patient’s perspective is ideal because they can most accurately report if/how counseling influenced their practices. Our sample was enrolled within one metropolitan area and the majority of subjects was of low socioeconomic status and thus may not be generalizable to all other substance dependent populations. We were unable to determine if patients received safe sex counseling from another type of clinician (other than their primary care doctor) or practiced safer sex because of advice from another clinician. There may also be temporal issues, in asking about lifetime safe sex counseling versus symptoms of mental illnesses, which may have manifested after counseling occurred (as described above).

We did not have data to account for the U.S. Preventive Services Task Force recommendations of counseling if patients had an STI within the last year, which may have affected safe sex counseling. Instead, we used number of sex partners in the last three months as an indicator of sexual risk behavior. We also used only partial diagnostic instruments as indicators of mental illness, particularly for psychotic symptoms. Despite these limitations, this study is among the first to examine racial/ethnic and mental health disparities in safe sex counseling and practices among individuals with substance dependence. This paper is one of many that will answer the call for studies to test hypotheses about how clinician behaviors affect disparities in health care [68]. Beyond merely documenting that counseling occurred, research needs to focus on enhancing our understanding of how counseling can have an impact on patient risk behavior.

## Conclusions

Black patients and those with a history of manic episodes were found to be counseled more about safe sex than white patients and those without manic episodes, respectively, despite controlling for sexual risk. Exploring the basis of how patient characteristics can influence counseling and resultant behavior merits further exploration to help reduce disparities in safe sex counseling and outcomes.

## Competing interests

The authors declare that they have no competing interests.

## Authors’ contributions

All authors have made substantial contributions to the study’s conception, design, data collection, analysis and/or interpretation of results, been involved in drafting or revising the manuscript and have approved the version to be published. *MMD* conceived of this study’s analysis plan, interpreted the results and drafted and revised the manuscript. *DMC* is a biostatistician and provided guidance in developing and revising the analytic plan, interpreting the results and revising the manuscript. *DAD* conducted the statistical programming for this study and revised the manuscript for publication. *JHS* was Principal Investigator of one of the grants that provided support for this study, made substantial contributions to the conception, design, data collection, analytic plan and data interpretation for this study and also repeatedly revised the manuscript. *RS* was Principal Investigator of one of the grants that provided support for this study, made substantial contributions to the conception, design, data collection, analysis and interpretation for this study and made multiple revisions to this manuscript.
